# Comparison of a pre-bariatric surgery very low-calorie ketogenic diet and the Mediterranean diet effects on weight loss, metabolic parameters, and liver size reduction

**DOI:** 10.1038/s41598-022-24959-z

**Published:** 2022-11-30

**Authors:** Nihal Zekiye Erdem, Demet Ozelgun, Halit Eren Taskin, Fatih Mehmet Avsar

**Affiliations:** 1 School of Health Sciences, Department of Nutrition and Dietetics, Istanbul Medipol University, Fatih, Istanbul Turkey; 2 Department of Nutrition and Dietetic, Institute of Health Sciences, Istanbul Medipol University, Beykoz, Istanbul Turkey; 3 Department of General Surgery, Cerrahpaşa Medical Faculty, Istanbul University Cerrahpaşa, Istanbul, Turkey; 4 Department of General Surgery, Health Sciences University, Gulhane Faculty of Medicine, Ankara City Health Practice and Research Center Hospital. Department of Surgical Oncology, Bilkent, Ankara Turkey

**Keywords:** Obesity, Outcomes research

## Abstract

This study compared the effects on weight as well as on metabolic parameters and liver size of a very low-calorie ketogenic diet versus a Mediterranean diet in patients with morbid obesity preparing to undergo bariatric surgery. This prospective comparison study evaluated patients 18–65 years of age who enrolled for bariatric surgery. Study duration was limited to an immediate preoperative period of 15 days. The very low-calorie ketogenic diet incorporated 10–12 kcal/kg/day of energy and 1–1.2 g/kg of protein using Kalibra (Societa Dietetica Medica) (VLCKD-SDM). The Mediterranean diet (MD) included 15–20% protein, 45–50% carbohydrate, and 25–35% fat. Changes in body mass index (BMI), liver size, and anthropometric and metabolic measurements were assessed. Between January 2016 and March 2017, of 45 patients enrolled, 30 completed the study (VLCKD-SDM, n = 15; MD, n = 15). Respective median BMI loss after VLCKD-SDM was 2.7 kg/m^2^ versus MD 1.4 kg/m^2^ (p < 0.05); median fat percentage reduction was 3.2 units versus 1.7 units (p < 0.05). Median liver size decreased 5.5% in the VLCKD-SDM group versus 1.7% in the MD group (p < 0.05). Median total cholesterol, and LDL levels decreased in both groups (p < 0.05), with greater relative decreases in the VLCKD-SDM group. Short-term preoperative diet-based weight loss in patients with morbid obesity preparing for bariatric surgery was significantly greater following a very low-calorie ketogenic diet versus a Mediterranean diet. The very low-calorie diet also significantly improved anthropometric and metabolic parameters and reduced preoperative liver size above that of the MD.

## Introduction

Obesity is a disease that affects approximately 641 million adults worldwide. Of these, 58 million men and 126 million women suffer from *morbid* obesity, which is typically associated with diseases such as non-alcoholic fatty liver disease (NAFLD), sleep apnea syndrome, hypertension, type 2 diabetes mellitus, and dyslipidemias^1^. Liver damage increases with increasing body mass index (BMI), and a large liver size is significantly related to increased surgical morbidity and mortality^2^. Prior to metabolic/bariatric surgery, intentional weight loss can successfully reduce liver size and improve intraoperative management of the anatomy. Short-term, low-energy, low-carbohydrate diets have been shown to be particularly effective in reducing BMI and liver size and in helping patients to anticipate postoperative weight loss^3–10^.

The efficacy of the Mediterranean diet (MD) in achieving weight loss in patients who are overweight or who suffer from obesity has been much publicized^9^. A variety of diets have been tested and compared with the MD, both longitudinally following bariatric surgery, and prior to surgery over a brief time period to induce rapid weight loss. Observational studies randomized controlled trials, and systematic reviews have cited the low-calorie ketogenic diet (VLCKD) as the most successful in achieving weight loss^2^. Ketogenic-based diets may induce greater weight loss by the expenditure of energy in gluconeogenesis due to the low glucose intake, decreased appetite, and increased energy expenditure resulting from ketosis^2,3^. In the current study, we assessed the effects of the MD versus a VLCKD undertaken by patients with morbid obesity over a 2-week pre-bariatric surgery time course. Weight loss is aimed to be the initial outcome of this study. It is intended to reduce the liver size by losing weight. An overall weight loss goal of 5–10% was thought to be safe and feasible, and has been associated with a reduction in visceral fat and liver size within a few weeks^5,11^. Preoperative changes in weight, liver size, anthropometric parameters, and metabolic outcomes for patients on each diet were compared.

## Methods

### Study design and approval

This prospective comparison study was undertaken to assess changes in morbidly obese patients in two groups, each undertaking a 2-week trial of a different diet before undergoing bariatric surgery. These diets are not routinely practiced in the clinic. This is not an interventional study. Patients were randomly assigned to diet types. The standard effect size was determined as 1.05 with 5% margin of error and 80% power for the sample size. It was found sufficient to include n = 14 cases in each group. According to this minimum number, 15 people were included in each group. The primary outcome used to base statistical power was body mass index. Forty-five morbidly obese patients were included in the study. At the beginning of the study before exclusion, 25 of these 45 patients were in the VLCKD-SDM group and 20 were in the MD group. After the excluded patients, the number of patients in each diet group fulfilled the numbers required for the sample size. Nine patients failed to adhere to the diet and were therefore excluded from the study. Of these patients, 7 were in the VLCKD-SDM and 2 were in the MD group. Additionally, 6 patients had early surgery. Three of these belonged to the VLCKD-SDM group, and three to the MD group. Patients who failed to follow the diet and underwent surgery too soon were unable to finish the 14-day diet phase. They were left out of the study to avoid having a negative impact on the findings. The study was conducted with pre-approval of the hospital’s Non-invasive Clinical Research Ethics Committee (Decision No. 161, Approval #10840098-604.01.01-E.12049 and E-10840098-772.02-2490; dated 09.03.2016, 29.07.2016, 01.06.2021) and was performed in accord with the ethical standards of the 1964 Declaration of Helsinki and its later amendments.

### Inclusion and exclusion

Patients seeking bariatric surgery who were 18–65 years of age with a BMI of ≥ 35.0 kg/m^2^ with up to two comorbidities were included in the study. Patients were excluded if they were females who were pregnant or lactating, or men and women with acute or chronic kidney failure (creatinine, women ≥ 1.2 mg/dL, men ≥ 1.3 mg/dL or glomerular filtration rate < 60–65 mL/min), chronic alcoholism, cancer, liver failure, acid–base balance disorder, severe psychiatric disorders, or active peptic ulcer disease.

### Diets

#### VLCKD-SDM

The very-low calorie ketogenic diet (VLCKD) group’s 2-week course consisted of meal replacements using Societa Dietetica Medica (SDM, Kalibra [VLCKD-SDM]) products. Patients were provided with 1–1.2 g/kg/day of protein based on each patient’s calculated ideal weight, 10–12 kcal/kg/day of energy (containing 30–40% fat) based on ideal weight. One portion of each product contained 16–18 g of protein (31 g, pasta, 20 g, focaccia), 60–224 kcal of energy, 0.1–12 g of fiber, and 0.9–9.6 g of fat. The respective protein content of the above products is composed of whey, egg, wheat, and pea protein. Products also contained important amino acids (i.e., leucine, lysine, isoleucine, valine, methionine, phenylalanine, threonine, and tryptophan) and sucralose approved by the U.S. Food and Drug Administration (FDA) as a sweetener.

In addition, patients were instructed to drink 2–3 L of water a day. Individualized nutritional supplements were given under the supervision of the doctor^12,13^. These included: 1 tablet Supradyn All Day (Bayer Turkish Chemical Industry Trade Ltd., Turkey); 1 effervescent tablet of Kalinor (Farma-Tek Pharmaceutical Industry, and Trade Co. Ltd., Germany); 2 tablets of calcium, Osteocare (Vitabiotics Ltd., UK); 1 capsule of n-3 polyunsaturated fatty acid (PUFA), Marincap (Kocak Farma Pharmaceutical, and Chemical Industry Trade Co. Ltd., Turkey); and 1 packet/day NBL Probiotic Gold (Cell Biotech Co. Ltd., South Korea).

#### Mediterranean diet

Based on an updated systematic review of the core compositional elements of a Mediterranean diet^14^, the study required patients in the MD group to consume 15–20% protein (low-fat dairy, poultry, fish, legumes, nuts, red meat) in their daily food intake, 45–50% carbohydrates (vegetables, fruits, whole grains), and 25–35% fat (especially from olive oil). In this MD, the daily minimum requirements of vitamins and minerals were expected to be included with the food and beverage selections; therefore, supplements were not added.

### Diet follow-up

Demographic information, anthropometric and biochemical measurements, liver size, and gastrointestinal issues were assessed carefully before and after the diet. An interview was conducted at the hospital outpatient clinic to evaluate the nutritional status of the patients in detail on the 7th and the 15th days after the start of the study. Dietary compliance, ketones in urine, and any complications were assessed.

### Determining food consumption status

The patients' required intake of energy and macronutrients for both diet groups was determined using a formula. Our ketogenic diet group was fed commercial products, therefore daily macronutrients were determined based on the information on the labels of the goods they consumed over the course of 14 days. A 24-h diary was kept by members of our Mediterranean diet group. Patients were instructed to record on a chart the foods and meals they consumed over the course of three days, one of which being a weekend. The amounts of all foods consumed were calculated using the units of g and kg. The portions of the stated contents were chosen based on standardised recipes^15^. Consumed foods and portion amounts of meals were one by one entered into the Nutrition Information System (BeBiS) database software programme. This software was used to compute the daily intake of energy, carbohydrate, fat, and micronutrients^16^.

### Evaluation of nutritional status

#### Anthropometric measurements

BMI was calculated using ideal BMI (IBMI) and ideal body weight (IBW) formulas^17,18^. Weight loss (WL%)^19^ and basal metabolic rate (BMR) were calculated with the Schofield Equation^20^. Body fat percentage and lean body mass (LBM) were calculated using standard formulas^21^. Waist, hip, and middle-upper arm circumference (MUAC) measurements were taken. Waist Circumference was assessed at the midpoint between the 12th rib and the iliac crest, while hip circumference was measured at the level of the greater trochanter. Waist/hip ratio was calculated as WC/HC^18,22^. Malnutrition status was determined by the nutritional risk index (NRI)^23^. Nutritional Risk Index (NRI) values were calculated by using plasma albumin levels and body weight alterations and compared with reference data NRI was calculated upon the following formula:

$$\mathrm{NRI }= 1.519 \times \mathrm{ Albumin }(\mathrm{g}/\mathrm{L}) \times 0.417 (\mathrm{Current weight}/\mathrm{Usual weight}) \times 100$$

NRI values were considered according to the data given below:

NRI > 100 normal,

NRI > 97.5 borderline malnutrition,

NRI = 83.5–97.5 moderate malnutrition,

NRI = 83.5 severe malnutrition.

#### Biochemical measurements

Biochemical measurements were performed after an overnight fast. These measurements were repeated two times in each group. Adherence to the protein-rich diet was detected by the presence of ketones in the urine.

#### Liver size

The liver size of patients was measured by the same radiologist in both study groups by abdominal sonography just before starting the pre-operative diet and 2 weeks after when the diet period ended just before the surgery. Both measurement values were assessed by the surgical team. No liver measurements were made in the postoperative period. The measurement was accomplished according to the method described by Börner et al., by taking the mid-clavicular line in maximum inspiration and supine positions as reference by using a 2–5-MHz convex abdominal sonography probe. Reference value, mean male ≤ 150 ± 15 mm and female ≤ 149 ± 16 mm values were considered normal^24^.

### Statistical evaluation

Analyses were performed using the SPSS statistical package (version 22, IBM, Chicago, IL, USA). Descriptive statistics were calculated for all variables assessed. Quantitative variables were reported as means, standard deviations (SDs), medians, minimums, and maximums. Qualitative variables were reported as frequencies and percentages. Interquartile ranges were used. Data normality was evaluated by visual inspection of histograms/Q–Q plots and cross-validated by the Kolmogorov Smirnov test. As appropriate, independent samples t-tests or Mann–Whitney U-tests were used to assess between-group differences with respect to quantitative baseline and outcome variables; chi-square tests (or Fisher's exact tests) were applied to qualitative data. Paired-samples t-tests or Wilcoxon signed-rank tests (as appropriate) were used to analyze within-group change from baseline along quantitative variables; McNemar’s test was used for qualitative data. Correlations of the independent variables were performed by Spearman's correlation tests. All statistical tests were two-tailed and statistical significance was set at p < 0.05.

### Ethical approval

Ethics Committee approval (# 10840098-604.01.01-E.12049, dated 09.03.2016 and 29.07.2016, Decision #161) was obtained to conduct the study from the Istanbul Medipol University Noninvasive Clinical Research Ethics Committee.

### Informed consent

Informed consent was obtained from all study participants.

### Human and animal rights

The study was performed in accord with the ethical standards of the 1964 Declaration of Helsinki and its subsequent amendments.

## Results

Between January 2016 and March 2017, 45 patients (25 in VLCKD-SDM, 20 in MD group) with morbid obesity between the ages of 18 and 65 years were enrolled in the General Surgery Department to undergo bariatric surgery. Nine patients (7 in VLCKD-SDM, 2 in MD group) could not adhere to the diet and 6 patients (3 in VLCKD-SDM, 3 in MD group) were operated on earlier than originally planned, excluding 15 patients from the study. Thus, the total study sample was 30 patients, 15 in the VLCKD-SDM group and 15 in the MD group. Each group consisted of 4 (26.7%) men and 11 (73.3%) women. The respective mean age of VLCKD-SDM versus MD patients was 42.9 ± 12.6 (median 46) years versus 41.3 ± 14.6 (median 47) years (p > 0.05); height, 160.3 ± 10.0 (median 158) cm versus 163.9 ± 8.2 (median 161) cm (p > 0.05); and absolute weight, 125.3 kg versus 135.0 kg.

The daily energy and macronutrients in each diet group are shown in Table 1.

**Table 1:** Daily intake of dietary energy and nutrients during the VLCKD-SDM and MD.

Dietary energy and nutrients	VLCKD-SDM (n = 15)		MD (n = 15)		p value*
	I.Q-3.Q	Median	I.Q-3.Q	Median	
Total energy (kcal)	600–700	650	1571–1870	1630	** < 0.001**
Carb. energy (kcal)	150.0–190.0	176	740.0–900.0	800	** < 0.001**
Carb. (g)	37.5–47.5	44	185.0–225.0	200	** < 0.001**
Carb. %	24.0–29.4	27.1	46.3–49.4	48.1	** < 0.001**
Protein (kcal)	240.0–280.0	240	300.0–340.0	300	** < 0.001**
Protein (g)	60.0–70.0	60	75.0–85.0	75	** < 0.001**
Protein (%)	37.1–40.0	38.5	18.1–19.4	18.9	** < 0.001**
Fat energy (kcal)	180.0–234.0	234	540.0–630.0	540	** < 0.001**
Fat (g)	20.0–26.0	26	60.0–70.0	60	** < 0.001**
Fat (%)	33.3–36.6	36	32.2–34.4	33.7	0.059

Significant values are in bold.

*N* number of the patients, *1.Q* interquartile range 25%, *3.Q* interquartile range 75%, *Carb*. carbohydrate.

*Mann–Whitney U test.

In conjunction with diet-induced weight loss, significant decreases in anthropometric measurements from baseline (p = 0.001) were observed in both groups (Table 2).

**Table 2:** Anthropometric measurements.

Anthropometric measurements	VLCKD-SDM (n = 15)		MD (n = 15)		p value*
	I.Q-3.Q	Median	I.Q-3.Q	Median	
IBMI (kg/m^2^)	22.0 to 24.0	24	22.0 to 24.0	24	0.563
IBW (kg)	54.7to 64.6	59.9	57.7 to 66.9	59.6	0.590
**Weight** (kg)					
Pre-diet	109.0 to 157.0	125.3	118.0 to 143.0	135	0.756
Post-diet	105.0 to 148.0	114	114.0 to 140.0	131	0.443
Change	− 9.0 to − 5.8	− 6.4	− 4.0 to − 3.0	− 3.3	** < 0.001**
***Change p***	**–**	** < 0.001**^§^	**–**	** < 0.001**^§^	
**Weight loss** **(%)**	4.8 to 6.7	5.4	2.1 to 3.1	2.5	** < 0.001**
**BMI (kg/m**^**2**^**)**					
Pre-diet	43.2 to 61.8	47.8	45.5 to 52.8	49.7	0.803
Post-diet	39.4 to 59.4	45.8	43.9 to 51.6	48.9	0.633
Change	− 3.3 to − 2.1	− 2.7	− 1.6 to − 1.0	− 1.4	** < 0.001**
***Change p***	**–**	** < 0.001**^§^	**–**	** < 0.001**^§^	
**NRI**	45.3 to 46.5	46.1	46.8 to 47.3	47	** < 0.001**
**MUAC (cm)**					
Pre-diet	38.0 to 47.0	42	41.0 to 47.0	43	0.505
Post-diet	34.0 to 44.0	38	40.0 to 46.0	42.5	**0.014**
Change	− 6.0 to − 2.0	− 3	− 1.0 to 0.0	− 0.5	** < 0.001**
***Change p***	**–**	** < 0.001**^§^	**–**	** < 0.001**^§^	
**Waist circumference (cm)**					
Pre-diet	123.0 to 150.0	135	127.0 to 150.0	140	0.803
Post-diet	113.0 to 141.0	130	124.0 to 146.0	136	0.361
Change	− 10.0 to − 5.0	− 8	− 4.0 to − 2.0	− 3	** < 0.001**
***Change p***	**–**	** < 0.001**^§^	**–**	** < 0.001**^§^	
**Hip circumference (cm)**					
Pre-diet	127.0 to 172.0	141	136.0 to 157.0	142	0.901
Post-diet	121.0 to 163.0	135	134.0 to 154.0	141	0.442
Change	− 11.0 to − 7.0	− 8	− 4.0 to − 1.0	− 2	** < 0.001**
***Change p***	**–**	** < 0.001**^§^	**–**	** < 0.001**^§^	
**Waist/hip ratio**					
Pre-diet	0.89 to 0.99	0.96	0.90 to 1.03	0.93	0.934
Post-diet	0.90 to 0.99	0.97	0.90 to 1.03	0.93	0.967
Change	− 0.01 to 0.02	0.01	− 0.01 to 0.01	− 0.01	0.633
***Change p***	**–**	** < 0.001**^§^	**–**	** < 0.001**^§^	
**Fat (%)**					
Pre-diet	52.8 to 71.1	56.2	52.4 to 69.4	58.6	0.934
Post-diet	50.3 to 67.6	53.3	51.5 to 67.6	57.4	0.663
Change	− 4.0 to − 2.5	− 3.2	− 1.9 to − 1.2	− 1.7	** < 0.001**
***Change p***	**–**	** < 0.001**^§^	**–**	** < 0.001**^§^	
**LBM (kg)**					
Pre-diet	54.3 to 86.3	57.5	58.8 to 74.0	67	0.633
Post-diet	46.4 to 79.9	53.5	55.9 to 72.0	65	0.419
Change	− 6.4 to − 4.1	− 4.6	− 2.8 to − 2.1	− 2.6	** < 0.001**
***Change p***	**–**	** < 0.001**^§^	**–**	** < 0.001**^§^	

Significant values are in bold.

*Mann–Whitney U test.

^†^Chi-square test (Fisher’s exact test).

^§^Wilcoxon test.

*N* number of the patients, *1.Q* interquartile range 25%, *3.Q* interquartile range 75%, *NRI* nutritional risk index, *MUAC* middle-upper arm circumference, *LBM* lean body mass.

Metabolic parameters in each diet group are shown in Table 3. The greater weight loss and changes in anthropometric measurements in the VLCKD-SDM group generally translated into statistically greater improvements in metabolic parameters (Table 3). Both diet groups experienced significant change from baseline in total cholesterol, low-density lipoprotein (LDL), and triglyceride (TG) levels (all p < 0.05); however, the reductions in the VLCKD-SDM group were significantly greater relative to the MD group with respect to total cholesterol, HDL, and LDL (all p < 0.05).

**Table 3:** Metabolic parameters.

Metabolic parameters	VLCKD-SDM (n = 15)		MD (n = 15)		p value*
	I.Q-3.Q	Median	I.Q-3.Q	Median	
**FBG (70–115 mg/dL)**					
Pre-diet	96.0 to 215.0	121	93.0 to 115.0	102	0.120
Post-diet	80.0 to 202.0	108	88.0 to 103.0	96	0.271
Change	− 50.0 to 1.0	− 13	− 12.0 to 4.0	− 5	0.198
***Change p***	–	0.051^†^	–	0.077^†^	
**Cholesterol (112–200 mg/dL)**					
Pre-diet	154.0 to 203.0	175	166.5 to 217.8	190	0.445
Post-diet	132.0 to 182.0	142	157.5 to 205.3	183.5	**0.012**
Change	− 34.0 to − 15.0	− 22	− 14.0 to − 4.0	− 0.6	**0.008**
***Change p***	–	** < 0.001**^†^	–	**0.004**^†^	
**HDL (28–75 mg/dL)**					
Pre-diet	35.0 to 54.0	44	37.8 to 54.3	47	0.555
Post-diet	33.0 to 46.0	39	38.0 to 48.3	45	0.175
Change	− 11.0 to − 3.0	− 5	− 6.0 to 2.0	− 3	**0.041**
***Change p***	–	**0.015**^†^	–	0.055^†^	
**LDL (0–160 mg/dL)**					
Pre-diet	90.0 to 132.0	112	93.0 to 156.0	129	0.361
Post-diet	74.0 to 110.0	96	94.3 to 153.5	130	**0.025**
Change	− 28.0 to − 10.0	− 16	− 8.0 to − 3.0	− 4.8	**0.003**
***Change p***	–	**0.004**^†^	–	**0.003**^†^	
**TG (35–150 mg/dL)**					
Pre-diet	102.0 to 213.0	114	86.0 to 233.0	156	0.983
Post-diet	94.0 to 177.0	108	80.3 to 208.3	129	0.727
Change	− 20.4 to − 7.0	− 11	− 21.0 to − 5.0	− 8	0.430
***Change p***	–	**0.009**^†^	–	**0.009**^†^	

Significant values are in bold.

*n* number of the patients, *1.Q* interquartile range 25%, *3.Q* interquartile range 75%, *FBG* fasting blood glucose, *HbA1C* Hemoglobin A1C, *HDL* high-density lipoprotein, *LDL* low-density lipoprotein, *TG* triglycerides.

*Mann–Whitney U test.

^†^Wilcoxon test.

Liver and blood biochemical parameters are shown in Table 4.

**Table 4:** Liver and blood biochemical parameters.

Biochemical measurements	VLCKD-SDM (n = 15)		MD (n = 15)		p value*
	I.Q-3.Q	Median	I.Q-3.Q	Median	
**Urea (10–50 mg/dL)**					
Pre-diet	22.0 to 34.0	25	21.0 to 37.0	30	0.755
Post-diet	21.0 to 39.0	28	19.0 to 34.0	31	0.967
Change	− 5.0 to 11.0	2	− 7.0 to 4.0	− 1	0.318
***Change p***	–	0.363^†^	–	0.660^†^	
**Uric acid** **(2–8 mg/dL)**					
Pre-diet	5.3 to 6.7	5.8	5.6 to 6.6	5.9	0.930
Post-diet	5.4 to 6.8	6.1	5.2 to 6.3	5.9	0.347
Change	− 0.2 to 0.4	+ 0.2	− 0.3 to 0.2	− 0.2	0.074
***Change p***	–	0.277^†^	–	0.098^†^	
**Creatinine** **(0.6–1.3 mg/dL)**					
Pre-diet	0.74 to 0.91	0.8	0.62 to 0.87	0.8	0.319
Post-diet	0.71 to 0.98	0.9	0.66 to 0.78	0.8	0.309
Change	− 0.07 to 0.09	0	− 0.11 to 0.12	0	0.430
***Change p***	–	0.609^†^	–	0.887^†^	
**Protein** **(6.4–8.3 g/dL)**					
Pre-diet	7.6 to 8.0	7.7	6.2 to 7.4	6.9	** < 0.001**
Post-diet	7.5 to 8.0	7.8	6.1 to 7.2	7.1	** < 0.001**
Change	− 0.2 to 0.3	0.21	− 0.3 to 0.2	− 0.08	0.170
***Change p***	–	0.118^†^	–	0.801^†^	
**Albumin** **(3.5–5.4 g/dL)**					
Pre-diet	3.8 to 4.5	4.2	4.1 to 4.6	4.3	0.382
Post-diet	4.1 to 4.6	4.2	4.1 to 4.5	4.2	0.575
Change	− 0.1 to 0.4	0.2	− 0.2 to 0.2	− 0.08	0.096
***Change p***	–	0.052^†^	–	0.729^†^	
**ALP** **(40–150 U/L)**					
Pre-diet	56.0 to 95.0	62	56.0 to 88.0	77	0.480
Post-diet	55.0 to 74.0	65	53.3 to 88.3	73.5	0.541
Change	− 7.0 to 4.0	− 4	− 8.0 to − 2.0	− 6	0.692
***Change p***	–	0.105^†^	–	0.052^†^	
**AST** **(5–34 U/L)**					
Pre-diet	19.0 to 25.0	22	15.0 to 32.0	21	0.803
Post-diet	23.0 to 37.0	31	16.0 to 31.0	27	0.236
Change	− 1.0 to 16.0	0	− 6.0 to 6.0	0	0.245
***Change p***	–	0.278^†^	–	0.850^†^	
**ALT** **(0–55 U/L)**					
Pre-diet	19.0 to 40.0	21	20.0 to 45.0	30	0.383
Post-diet	21.0 to 50.0	36	20.0 to 44.0	29	0.575
Change	− 2.0 to 19.8	2	− 7.4 to 1.0	− 1	0.129
***Change p***	–	0.300^†^	–	0.180^†^	
**Serum Iron** **(25–156 μg/dL**)					
Pre-diet	63.0 to 106.0	87	46.0 to 94.0	67	0.184
Post-diet	62.0 to 119.0	94	47.5 to 99.8	79	0.206
Change	2.0 to 11.0	8	− 2.0 to 11.0	7	0.851
***Change p***	–	0.053^†^	–	0.076^†^	
**Serum iron binding (110–370 μg/dL)**					
Pre-diet	211.0 to 332.0	269	224.0 to 336.8	288.5	0.600
Post-diet	174.0 to 308.0	243	212.0 to 342.8	283	0.471
Change	− 26.0 to − 11.0	− 15	− 14.0 to 4.0	− 11	**0.049**
***Change p***	–	**0.009**^†^	–	0.157^†^	
**CRP** **(0.001–5 mg/dL)**					
Pre-diet	2.1 to 15.0	6	1.8 to 5.1	3.2	**0.041**
Post-diet	1.6 to 16.0	5.2	1.8 to 4.1	2.2	**0.048**
Change	− 2.6 to − 0.2	− 0.6	− 0.8 to − 0.2	− 0.7	0.319
***Change p***	**–**	**0.031**^†^	**–**	**0.007**^†^	
**B12** **(145–980 pg/mL)**					
Pre-diet	158.0 to 306.0	214	169.8 to 325.3	255.5	0.485
Post-diet	186.0 to 385.6	258	186.3 to 326.3	251	0.647
Change	12.0 to 44.0	18	− 3.0 to 18.0	12	0.041
***Change p***	–	** < 0.001**^†^	–	0.187^†^	
**D-Vit** **(ng/mL)**					
Pre-diet	7.1 to 12.0	8.2	7.7 to 13.0	10.5	0.445
Post-diet	9.2 to 21.1	11	8.3 to 14.8	11.4	0.256
Change	1.6 to 5.4	2.1	0.2 to 1.2	0.7	** < 0.001**
***Change p***	–	** < 0.001**^†^	–	**0.002**^†^	
**Ferritin (5–148 ng/mL)**					
Pre-diet	62.0 to 127.0	87	78.0 to 118.8	93	0.585
Post-diet	64.0 to 142.0	95	66.5 to 125.8	92	0.743
Change	− 21.0 to 15.0	6	− 13.0 to 5.0	− 4	0.213
***Change p***	–	0.798^†^	–	0.084^†^	
**Liver volume** **(mm)**					
Pre-diet	171.0 to 210.0	200	175.0 to 199.0	181	0.280
Post-diet	160.0 to 196.0	185	171.0 to 190.0	179	0.755
Change	− 15.0 to − 7.0	− 11.0 (5.5%)	− 4.0 to − 2.0	− 3.0 (1.7%)	** < 0.001**
***Change p***	–	** < 0.001**^**†**^	–	**0.008**^**†**^	

Significant values are in bold.

*N* number of the patients, *1.Q* interquartile range 25%, *3.Q* interquartile range 75%, *ALP* alkaline phosphatase, *ALT* aspartate aminotransferase, *AST* alanine aminotransferase, *CRP* C-reactive protein.

*Mann–Whitney U test.

^†^Wilcoxon test.

Finally, urinalysis revealed ketone presence in all VLCKD-SDM patients; however, this was not the case for all patients in the MD group. The McNemar test was used to statistically evaluate the ketone measurement in the urine.

Correlations between total energy, weight loss%, fat%, and LBM and metabolic parameters from Total Group (VLCKD-SDM + MD), VLCKD-SDM and MD are shown in Addendum Tables 1, X2, C3.

Minor complications of the two diets included nausea, constipation, and diarrhea in the VLCKD-SDM group, and only constipation in the MD group.

## Discussion

The protective effects on the cardiovascular system of implementing a long-term Mediterranean diet have been well demonstrated. In the 1960s, Ancel Keys formally elaborated the characteristics of the MD^25^ as inclusive of monosaturated fats (especially from olive oil), vegetables and fruits, whole grains, and low-fat dairy consumed daily; poultry, fish, legumes and nuts consumer weekly; and modest consumption of red meat 2–3 times monthly. Epidemiological studies and clinical trials have reported the anti-metabolic syndrome effects and anti-inflammatory and antioxidant properties of implementing a MD^26–28^. More recently, VLCKDs, MDs, and other programs comprised of appropriate protein and energy levels have demonstrated statistically significant preoperative weight loss and improvement of anatomical access after short-term pre-bariatric surgery diets.

The literature reports an array of preoperative dietary program durations, ranging from 10 days through 20 weeks, and a variety of claims regarding the reduction of intraoperative risks, postoperative complications, operating room time, hospital stay, and improvement of comorbidities through preoperative dieting^7,29^. Our discussion herein focuses solely on comparisons of our findings with recent studies of diets of between 10 days and 12 weeks’ duration, and on changes in weight loss, liver size reduction, and metabolic parameters^30–32^. We believe the current study is the first to directly compare an MD with a VLCKD product for a duration of 2 weeks to identify which approach facilitated the most advantageous overall preoperative weight loss in patients with morbid obesity.

The specific VLCKD in our study, VLCKD-SDM, contained n-3 PUFA, whey protein, and probiotic and vitamin/mineral supplements, and the MD was based on normal foods with carefully prescribed content and measures. It was comprised of an amount of protein similar to that of other VLCKDs, with a greater amount of fat^3,29,33^. The program consisted of industrial foods with whey protein, with a level of carbohydrate 17–29% higher than that of other VLCKDs and 18–20% lower than in other VLCDs and LCDs. On the other hand, the Mediterranean diet we studied can only be discussed in the context of the Pro-MD product identified in the literature. The MD diet included protein and fat content that was 8–12% higher than that of Pro-MD^9^.

The short-term weight loss goal of 5–10% was thought to be safe and feasible. Patients on the VLCKD-SDM attained this goal fully; MD patients achieved 50.0% of this primary weight loss endpoint. The VLCKD-SDM resulted in significantly greater decreases in LDL, and HDL compared to the MD, and, significantly greater increases in vitamin B12 and D levels. VLCKD-SDM also achieved a greater reduction in median liver size (5.5% versus 1.7%). In our study, as in other studies, serum iron levels trended toward significant increases with the duration of diet therapy but remained within normal limits^3^. We did not assess the effect of the two diets on complications, as systematic reviews suggest that successful preoperative weight-loss diets do not necessarily confer protection against intraoperative risks or postoperative complications^8^.

Other presurgical diets are based predominantly on meal replacements consisting of whey-casein proteins with added micronutrients and macronutrients. Diets of this type include: industrial liquid formulations of VLCD, 400–800 kcal/day for 10 days–12 weeks^2,4,7,30,31^; normal food-based VLCDs, 400–800 kcal/day for 10 days–6 weeks^3,6,18,32,33^; industrial liquid formulations of LCDs, 800–1500 kcal/day for 3–7 weeks^2,34–36^; normal/industrial food-based LCDs, for 10 days–4 weeks (3,36); protein-based Mediterranean diets (Protein-MD), 1200 kcal/day for 8 weeks^9^; and very low-calorie ketogenic diets (VLCKD), 400–800 kcal/day for 10–30 days)^3,29,33,37,38^.

Recent short-term presurgical studies, randomized trials, and a systematic review provide a basis for estimating the relative effectiveness of VLCDs, industrial liquid formulations of VLCDs, normal food-based VLCDs, industrial liquid formulations of VLCDs, a Protein-MD, and VLCKDs on weight, BMI, waist circumference, fat percentage, lean body mass, nutrient content, triglycerides, high-density lipoprotein (HDL), and liver size^2–12,14,27–39^. As depicted in the reference Protein-MD and VLCKDs reviewed were equivalent to one another in achieving the greatest percentage fat loss and liver size reduction; VLCKDs were also most effective in increasing lean body mass. The reference Protein-MD resulted in the greatest weight loss and HDL reduction. The reference VLCKD-SDMs were dominant in reducing BMI and waist circumference. Essential nutrients were most increased by the reference industrial liquid formulations VLCDs; and triglycerides were most decreased by normal food based VLCDs.

No nutritional supplements were administered since MD contains a significant amount of nutrients and is balanced and healthy. Vitamins and probiotics were added to the ketogenic commercial meals consumed in VLCKD-SDM as they lacked appropriate vitamin and fiber content. With the nutritional supplements given to the ketogenic diet group, it was made sure that the two diet groups in this study had comparable ratios of all macronutrients, with the exception of protein and carbohydrates.

The contents of the two meals were assessed for these nutritional supplements, but whether they were in the same quantities was not determined. Although no calculations were conducted, it is believed that both groups' nutrients are relatively similar. The outcomes demonstrate this as well (Table 4). It was discovered that there were no statistically significant variations between these two diet groups' biochemical markers. In VLCKD-SDM, only vitamin B12 and vitamin D levels increased. This also has to do with supplements. High levels of vit B12 and D in VLCKD are thought to be related to supplementation. Patients on the MD diet did not receive vit D supplements according to the pre-diet blood levels of vit D. The impact on the biochemical outcomes might have been the same if MD had received B12 and vitamin D supplements in accordance with the levels of vitamin D in the blood. According to studies in the literature, patients' constipation and elevated ketone levels are caused by ketogenic industrial items. Based on these data, probiotic supplementation was administered. Fish eating is part of the healthy MD nutritional approach, hence was presented in MD. Patients with VLCKD-SDM were supplemented with n-3 PUFAs since fish could not be provided to them. Potassium was given in studies with VLCKD. This was given because it was a consensus. To ensure that the nutritional profiles of the two groups were comparable, n-3 PUFA supplementation was given to the VLCKD-SDM group in this study. They were not supplied separately as supplements since it was believed that these nutrients were satisfied by natural meals in the MD group. Therefore, giving n-3 PUFA supplements to VLCKD-SDM does not completely neutralize this superiority of MD.

In relating current results to that of the reference diets in terms of duration, the mean weight loss attained by patients in the 2-week VLCKD-SDM (7.3 ± 2.3 kg, total weight loss [TWL] 5.7%) was reached only by the reference industrial liquid formulations VLCDs and industrial liquid formulations of LCDs following 3–6 weeks. Weight loss achieved in the studied MD (3.4 ± 0.8 kg, TWL 2.6%) was approximately half that of the reference VLCKDs, more than half of the industrial liquid formulations VLCDs and industrial liquid formulations of LCDs, and equivalent to the normal food-based VLCDs and normal/industrial food-based LCDs.

The decrease in HDL levels was greater in VLCKD-SDM. Both total and VLCKD-SDM showed a positive correlation between post-diet HDL change and LBM levels (i.e., as HDL levels decreased, LBM levels also decreased, or vice versa) (Addendum Table 2). In the total group, a positive correlation was observed between post-diet HDL change and fat% and weight loss% (Addendum Table 1). It was observed that HDL decreased more with increasing weight loss, which is consistent with the literature^3,6,40^. As in the article, during the acute phase of weight loss, HDL levels fall as a result of a change in the activity of lecithin cholesterol ester transfer protein (LCAT), and when low body weight is stably maintained, HDL either rises to baseline levels or higher^40^. During weight loss therapy using VLCDs, HDL initially decreases, but then either increases to baseline levels or results in an overall improvement in HDL levels during weight maintenance^3,6,40^.

Reference diets, as a group, appeared to reduce liver size by between 12 and 30%. A systematic review of preoperative diets showed that liver size decreased on average by 15% (range 5–20%), though not with low-energy diets^8^. The greatest reduction in liver size among the reference diets was in the Protein-MD group and VLCKD group between 2 and 3 weeks. In VLCKD studies, the occurrence of ketonuria and ketonemia is evidence of compliance with the diet, wherein a 1-unit increase in ketonuria increased TWL by 2.0%^3,6,37^. In this context, the greater weight loss seen in the studied VLCKD-SDM group versus the MD group was likely associated with a higher satiety level related to the development of ketones.

## Limitations

Limitations of our study included a relatively low number of patients, the short-term nature of the study, and the difficulty of comparing the studied diets to other reported diets of varying durations. Dividing the VLCKD-SDM group into two subgroups (one containing added n-3, one without it) would have provided additional insight into the most effective form of this diet.

The limitations of the study include that nutrients were not calculated in the same ratios in both groups before the diet, and supplements were not administered to our MD group based on pre-diet serum vitamin-mineral levels. Further research should be conducted by administering supplements in the same ratio to both groups.

## Conclusions

Ketogenic diets have higher protein and lower carbohydrate content than MDs, and for this reason may be the superior preoperative 2-week weight-loss diet. The MD appears to require a significantly longer period of time to achieve similar results. The MD facilitated 50.0% less weight loss than the VLCKD-SDM. However, if the VLCKD-SDM results in gastrointestinal side effects, the MD can be effectively employed.

To the best of our knowledge, this is the first study to directly compare the preoperative effects of a VLCKD with a Mediterranean diet. The current findings showed that liver size was significantly decreased following both diets; however, the VLCKD-SDM, with its high whey protein content, sufficient pre-measured energy, n-3 PUFA, and a balance of macro and micro nutrients appeared significantly more effective than the Mediterranean diet in reducing weight prior to bariatric surgery.

## Supplementary Information

Supplementary Table 1.

Supplementary Table 2.

Supplementary Table 3.

## Data Availability

Raw data pertaining to this study are not publicly available outside of the university database for ethical reasons with the exception of its presentation in this analyzed article for publication in the medical literature. The data that support the findings of this study are openly available by reasonable request to the first author (Dr. Nihal Zekiye Erdem: nzerdem@yahoo.com).
