# Supplementary material for: Comparison of a pre-bariatric surgery very low-calorie ketogenic diet and the Mediterranean diet effects on weight loss, metabolic parameters, and liver size reduction
Source: Sci Rep. 2022 Nov 30;12:20686. doi: 10.1038/s41598-022-24959-z (PMC9712493; doi:10.1038/s41598-022-24959-z)
Supplement: Supplementary file 1 — Supplementary Table 1. [file 41598_2022_24959_MOESM1_ESM.docx]

| **Total Group (VLCKD-SDM+MD)** | | **Total Energy** | |  | **Weight loss (%)** | |  | **Fat %** | |  | **LBM** | |
| --- | --- | --- | --- | --- | --- | --- | --- | --- | --- | --- | --- | --- |
|  |  | **r** | **p** |  | **r** | **p** |  | **r** | **p** |  | **r** | **p** |
| Total Energy | |  |  |  | 0.043 | 0.823 |  |  |  |  |  |  |
| Fat % | Pre-Diet | 0.161 | 0.397 |  | -0.176 | 0.352 |  |  |  |  |  |  |
|  | Post-Diet | 0.165 | 0.382 |  | -0.252 | 0.179 |  |  |  |  |  |  |
|  | Change | -0.208 | 0.269 |  | 0.920 | ***0.001*** |  |  |  |  |  |  |
| LBM | Pre-Diet | -0.007 | 0.971 |  | -0.048 | 0.801 |  | 0.572 | ***0.001*** |  |  |  |
|  | Post-Diet | -0.029 | 0.878 |  | -0.142 | 0.456 |  | 0.603 | ***0.001*** |  |  |  |
|  | Change | -0.089 | 0.641 |  | 0.960 | ***0.001*** |  | -0.023 | 0.904 |  |  |  |
| FBG | Pre-Diet | -0.179 | 0.343 |  | 0.235 | 0.211 |  | -0.107 | 0.575 |  | 0.234 | 0.214 |
|  | Post-Diet | 0.140 | 0.460 |  | 0.026 | 0.890 |  | 0.154 | 0.418 |  | 0.311 | 0.095 |
|  | Change | 0.318 | 0.087 |  | 0.333 | 0.072 |  | 0.204 | 0.281 |  | 0.272 | 0.147 |
| Cholesterol | Pre-Diet | -0.077 | 0.690 |  | -0.074 | 0.704 |  | 0.023 | 0.906 |  | 0.279 | 0.143 |
|  | Post-Diet | 0.156 | 0.418 |  | -0.406 | ***0.029*** |  | 0.108 | 0.577 |  | 0.140 | 0.468 |
|  | Change | 0.129 | 0.497 |  | 0.496 | ***0.005*** |  | 0.393 | ***0.032*** |  | 0.477 | ***0.008*** |
| HDL | Pre-Diet | 0.053 | 0.783 |  | 0.019 | 0.923 |  | 0.139 | 0.471 |  | 0.354 | 0.059 |
|  | Post-Diet | 0.277 | 0.146 |  | -0.115 | 0.554 |  | 0.059 | 0.760 |  | 0.235 | 0.221 |
|  | Change | 0.091 | 0.631 |  | 0.380 | ***0.038*** |  | 0.420 | ***0.021*** |  | 0.430 | ***0.018*** |
| LDL | Pre-Diet | 0.150 | 0.428 |  | -0.083 | 0.663 |  | 0.217 | 0.249 |  | 0.215 | 0.254 |
|  | Post-Diet | 0.181 | 0.348 |  | -0.250 | 0.190 |  | 0.322 | 0.089 |  | 0.265 | 0.164 |
|  | Change | 0.167 | 0.377 |  | 0.630 | ***0.001*** |  | 0.480 | ***0.007*** |  | 0.581 | ***0.001*** |
| TG | Pre-Diet | -0.101 | 0.597 |  | -0.056 | 0.769 |  | -0.278 | 0.136 |  | -0.226 | 0.231 |
|  | Post-Diet | -0.015 | 0.939 |  | -0.083 | 0.670 |  | -0.180 | 0.350 |  | -0.245 | 0.199 |
|  | Change | 0.072 | 0.707 |  | 0.060 | 0.752 |  | -0.054 | 0.777 |  | 0.031 | 0.869 |

**Addendum Table 1.** Correlations between total energy (of the two diets), weight loss%, fat% and LBM and metabolic parameters

Spearman Correlation.

LBM, lean body mass; FBG, fasting blood glucose; HDL, high-density lipoprotein; LDL, low-density lipoprotein; TG, triglycerides.
