# Supplementary material for: Comparison of a pre-bariatric surgery very low-calorie ketogenic diet and the Mediterranean diet effects on weight loss, metabolic parameters, and liver size reduction
Source: Sci Rep. 2022 Nov 30;12:20686. doi: 10.1038/s41598-022-24959-z (PMC9712493; doi:10.1038/s41598-022-24959-z)
Supplement: Supplementary file 2 — Supplementary Table 2. [file 41598_2022_24959_MOESM2_ESM.docx]

| **VLCKD-SDM Group** | | **Total Energy** | |  | **Weight loss (%)** | |  | **Fat %** | |  | **LBM** | |
| --- | --- | --- | --- | --- | --- | --- | --- | --- | --- | --- | --- | --- |
|  |  | **r** | **p** |  | **r** | **p** |  | **r** | **p** |  | **r** | **p** |
| Total Energy | | 1.000 |  |  | -0.431 | 0.109 |  |  |  |  |  |  |
| Fat % | Pre-Diet | 0.107 | 0.704 |  | -0.177 | 0.528 |  |  |  |  |  |  |
|  | Post-Diet | 0.184 | 0.511 |  | -0.245 | 0.379 |  |  |  |  |  |  |
|  | Change | 0.260 | 0.350 |  | 0.642 | ***0.010*** |  |  |  |  |  |  |
| LBM | Pre-Diet | -0.091 | 0.748 |  | -0.145 | 0.606 |  | 0.496 | 0.060 |  |  |  |
|  | Post-Diet | -0.046 | 0.871 |  | -0.163 | 0.562 |  | 0.506 | 0.054 |  |  |  |
|  | Change | 0.341 | 0.214 |  | 0.771 | ***0.001*** |  | -0.335 | 0.223 |  |  |  |
| FBG | Pre-Diet | -0.381 | 0.161 |  | -0.054 | 0.849 |  | -0.214 | 0.443 |  | 0.408 | 0.132 |
|  | Post-Diet | 0.199 | 0.478 |  | -0.309 | 0.262 |  | 0.120 | 0.670 |  | 0.424 | 0.115 |
|  | Change | 0.730 | ***0.002*** |  | 0.229 | 0.412 |  | -0.148 | 0.597 |  | 0.005 | 0.985 |
| Cholesterol | Pre-Diet | -0.013 | 0.965 |  | 0.123 | 0.661 |  | -0.034 | 0.904 |  | 0.327 | 0.234 |
|  | Post-Diet | 0.505 | 0.055 |  | 0.005 | 0.985 |  | 0.032 | 0.909 |  | 0.054 | 0.849 |
|  | Change | 0.367 | 0.178 |  | 0.145 | 0.606 |  | 0.086 | 0.761 |  | 0.156 | 0.579 |
| HDL | Pre-Diet | -0.120 | 0.671 |  | 0.250 | 0.369 |  | -0.022 | 0.937 |  | 0.239 | 0.391 |
|  | Post-Diet | 0.250 | 0.368 |  | 0.043 | 0.879 |  | -0.041 | 0.884 |  | 0.177 | 0.527 |
|  | Change | 0.484 | 0.067 |  | 0.452 | 0.091 |  | 0.412 | 0.127 |  | 0.576 | ***0.025*** |
| LDL | Pre-Diet | -0.058 | 0.839 |  | 0.163 | 0.562 |  | -0.039 | 0.889 |  | 0.156 | 0.580 |
|  | Post-Diet | 0.253 | 0.363 |  | 0.168 | 0.549 |  | 0.245 | 0.378 |  | 0.272 | 0.326 |
|  | Change | 0.386 | 0.155 |  | 0.203 | 0.467 |  | -0.062 | 0.827 |  | 0.074 | 0.792 |
| TG | Pre-Diet | 0.199 | 0.476 |  | -0.234 | 0.401 |  | -0.181 | 0.520 |  | 0.030 | 0.914 |
|  | Post-Diet | 0.176 | 0.532 |  | 0.070 | 0.805 |  | -0.064 | 0.819 |  | 0.000 | 1.000 |
|  | Change | -0.264 | 0.341 |  | 0.628 | ***0.012*** |  | -0.512 | 0.051 |  | -0.558 | ***0.031*** |

**Addendum Table 2.** Correlations between total energy, weight loss%, fat% and LBM and metabolic parameters from VLCKD-SDM

Spearman Correlation.

LBM, lean body mass; FBG, fasting blood glucose; HDL, high-density lipoprotein; LDL, low-density lipoprotein; TG, triglycerides.
