# Supplementary material for: Comparison of a pre-bariatric surgery very low-calorie ketogenic diet and the Mediterranean diet effects on weight loss, metabolic parameters, and liver size reduction
Source: Sci Rep. 2022 Nov 30;12:20686. doi: 10.1038/s41598-022-24959-z (PMC9712493; doi:10.1038/s41598-022-24959-z)
Supplement: Supplementary file 3 — Supplementary Table 3. [file 41598_2022_24959_MOESM3_ESM.docx]

**Addendum Table 3.** Correlations between total energy, weight loss%, fat% and LBM from MD and metabolic parameters

| **MD Group** | | **Total Energy** | |  | **Weight loss (%)** | |  | **Fat %** | |  | **LBM** | |
| --- | --- | --- | --- | --- | --- | --- | --- | --- | --- | --- | --- | --- |
|  |  | **r** | **p** |  | **r** | **p** |  | **r** | **p** |  | **r** | **p** |
| Total Energy | | 1.000 |  |  | 0.027 | 0.923 |  |  |  |  |  |  |
| Fat % | Pre-Diet | 0.264 | 0.341 |  | -0.307 | 0.265 |  |  |  |  |  |  |
|  | Post-Diet | 0.264 | 0.341 |  | -0.307 | 0.265 |  |  |  |  |  |  |
|  | Change | -0.396 | 0.144 |  | 0.775 | ***0.001*** |  |  |  |  |  |  |
| LBM | Pre-Diet | 0.064 | 0.821 |  | -0.154 | 0.585 |  | 0.539 | ***0.038*** |  |  |  |
|  | Post-Diet | 0.071 | 0.801 |  | -0.207 | 0.459 |  | 0.571 | ***0.026*** |  |  |  |
|  | Change | -0.095 | 0.735 |  | 0.908 | ***0.001*** |  | 0.865 | ***0.001*** |  |  |  |
| FBG | Pre-Diet | 0.126 | 0.655 |  | -0.096 | 0.732 |  | 0.014 | 0.960 |  | -0.079 | 0.781 |
|  | Post-Diet | 0.087 | 0.759 |  | -0.270 | 0.331 |  | 0.368 | 0.177 |  | 0.236 | 0.397 |
|  | Change | -0.177 | 0.528 |  | 0.256 | 0.356 |  | 0.173 | 0.537 |  | 0.262 | 0.345 |
| Cholesterol | Pre-Diet | 0.056 | 0.848 |  | 0.095 | 0.748 |  | 0.059 | 0.840 |  | 0.103 | 0.725 |
|  | Post-Diet | -0.023 | 0.939 |  | -0.077 | 0.794 |  | 0.106 | 0.719 |  | 0.198 | 0.497 |
|  | Change | 0.101 | 0.721 |  | 0.251 | 0.366 |  | 0.054 | 0.849 |  | 0.191 | 0.495 |
| HDL | Pre-Diet | 0.327 | 0.254 |  | 0.243 | 0.403 |  | 0.236 | 0.417 |  | 0.470 | 0.090 |
|  | Post-Diet | 0.468 | 0.092 |  | 0.483 | 0.080 |  | 0.150 | 0.609 |  | 0.282 | 0.328 |
|  | Change | -0.173 | 0.537 |  | 0.289 | 0.296 |  | -0.065 | 0.819 |  | -0.174 | 0.534 |
| LDL | Pre-Diet | 0.386 | 0.155 |  | 0.168 | 0.549 |  | 0.390 | 0.151 |  | 0.164 | 0.558 |
|  | Post-Diet | 0.277 | 0.337 |  | 0.305 | 0.288 |  | 0.319 | 0.267 |  | 0.147 | 0.615 |
|  | Change | 0.152 | 0.589 |  | 0.526 | ***0.044*** |  | 0.349 | 0.202 |  | 0.479 | 0.071 |
| TG | Pre-Diet | -0.266 | 0.337 |  | 0.018 | 0.950 |  | -0.436 | 0.104 |  | -0.482 | 0.069 |
|  | Post-Diet | -0.068 | 0.818 |  | -0.165 | 0.573 |  | -0.292 | 0.311 |  | -0.525 | 0.054 |
|  | Change | 0.479 | 0.071 |  | 0.382 | 0.160 |  | 0.000 | 1.000 |  | 0.244 | 0.381 |

Spearman Correlation.

LBM, lean body mass; FBG, fasting blood glucose; HDL, high-density lipoprotein; LDL, low-density lipoprotein; TG, triglycerides.
